# Usability Study of Mainstream Wearable Fitness Devices: Feature Analysis and System Usability Scale Evaluation

**DOI:** 10.2196/11066

**Published:** 2018-11-08

**Authors:** Jun Liang, Deqiang Xian, Xingyu Liu, Jing Fu, Xingting Zhang, Buzhou Tang, Jianbo Lei

**Affiliations:** 1 IT Center Second Affiliated Hospital, School of Medicine, Zhejiang University Hangzhou China; 2 College of Information Engineering China Jiliang University Hangzhou China; 3 Sichuan College of Traditional Chinese Medicine Mianyang China; 4 Affiliated Hospital of Stomatology, Southwest Medical University Luzhou China; 5 International School Southwest Medical University Luzhou China; 6 Peking University Third Hospital Beijing China; 7 Shenzhen HIT Campus Harbin Institute of Technology Shenzhen China; 8 Center for Medical Informatics Peking University Beijing China; 9 School of Medical Informatics and Engineering Southwest Medical University Luzhou China

**Keywords:** wearable devices, usability, System Usability Scale, function comparison, fitness

## Abstract

**Background:**

Wearable devices have the potential to promote a healthy lifestyle because of their real-time data monitoring capabilities. However, device usability is a critical factor that determines whether they will be adopted on a large scale. Usability studies on wearable devices are still scarce.

**Objective:**

This study aims to compare the functions and attributes of seven mainstream wearable devices and to evaluate their usability.

**Methods:**

The wearable devices selected were the Apple Watch, Samsung Gear S, Fitbit Surge, Jawbone Up3, Mi Band, Huawei Honor B2, and Misfit Shine. A mixed method of feature comparison and a System Usability Scale (SUS) evaluation based on 388 participants was applied; the higher the SUS score, the better the usability of the product.

**Results:**

For features, all devices had step counting, an activity timer, and distance recording functions. The Samsung Gear S had a unique sports track recording feature and the Huawei Honor B2 had a unique wireless earphone. The Apple Watch, Samsung Gear S, Jawbone Up3, and Fitbit Surge could measure heart rate. All the devices were able to monitor sleep, except the Apple Watch. For product characteristics, including attributes such as weight, battery life, price, and 22 functions such as step counting, activity time, activity type identification, sleep monitoring, and expandable new features, we found a very weak negative correlation between the SUS scores and price (*r*=−.10, *P*=.03) and devices that support expandable new features (*r*=−.11, *P*=.02), and a very weak positive correlation between the SUS scores and devices that support the activity type identification function (*r*=.11, *P*=.02). The Huawei Honor B2 received the highest score of mean 67.6 (SD 16.1); the lowest Apple Watch score was only 61.4 (SD 14.7). No significant difference was observed among brands. The SUS score had a moderate positive correlation with the user’s experience (length of time the device was used) (*r*=.32, *P*<.001); participants in the medical and health care industries gave a significantly higher score (mean 61.1, SD 17.9 vs mean 68.7, SD 14.5, *P*=.03).

**Conclusions:**

The functions of wearable devices tend to be homogeneous and usability is similar across various brands. Overall, Mi Band had the lowest price and the lightest weight. Misfit Shine had the longest battery life and most functions, and participants in the medical and health care industries had the best evaluation of wearable devices. The perceived usability of mainstream wearable devices is unsatisfactory and customer loyalty is not high. A consumer’s SUS rating for a wearable device is related to their personal situation instead of the device brand. Device manufacturers should put more effort into developing innovative functions and improving the usability of their products by integrating more cognitive behavior change techniques.

## Introduction

### Background

In recent years, wearable devices have gained tremendous momentum and have become part of people’s daily lives. Wearable devices are portable mobile intelligent devices that can be worn directly or integrated into clothing [1]. The wearable devices in this study are wristbands and watches with step counting as their core function [2]. After combining IMS market research consulting with 2012 market research data from ABI Research, the US market research firm BI Intelligence believed that less than 40% of US consumers were familiar with wearable devices in 2012, whereas at least 54% of consumers developed a strong interest in them for health monitoring and fitness tracking in 2013. BI Intelligence expects the global wearable market to grow at a compound annual rate of 35% between 2015 and 2019 [3]. According to data from the International Data Corporation’s Worldwide Quarterly Wearable Device Tracker, worldwide shipments of wearable devices grew by 1.2% in the first quarter of 2018 [4]. In addition, global smartwatch shipments in the second quarter of 2018 grew by 56% year on year [5]. Meanwhile, based on their original wearable market forecast from 2013 and interviews with industry experts, BI Intelligence concluded that, based on current trends, at least 33% of the US population will own wearable devices by the second quarter of 2017 [6]. The Tencent ISUX User Research Center released a report (smart wearable equipment market white paper) in 2015 [7] that contained a large-scale questionnaire survey with a total of 8083 domestic Chinese Tencent Instant Messenger user respondents. This stated that approximately 60% of internet users were familiar with wearable devices and that the number of Chinese internet users who owned a wearable device increased from 2.9% to 8.4% between November 2014 and May 2015. Among these users, wristband ownership was 4.6%, which was significantly higher than the 3.1% ownership of intelligent watches. Awareness of intelligent watches increased from 48% to 52%, and awareness of intelligent wristbands increased from 35% to 40% over the same period.

Wearable devices have significant potential for health monitoring and they are being widely investigated [8-13]. As a very popular tool, the use of wearable devices in medical and fitness applications is rapidly increasing. These devices can monitor vital signs such as motion [14], nutrition status [15], and heart rate [16], as well as a diabetic patient’s blood sugar [17], cardiac disease status, apnea during sleep, and numerous other health parameters and statuses [18]. Luo and Fan [19] believed that the development of wearable devices may trigger innovation in medical treatment models. Kirk [20] believed that wearable medical technology may significantly improve the curative effect for patients, reduce physician time, and lower medical costs, representing future trends of precise personalized medical treatment. Appropriate technology, such as smart wearable devices, may create unprecedented opportunities for medical and health care service providers to obtain the accurate real-time data required to prevent, diagnose, and treat various chronic diseases in a more economical manner [21]. Recent studies have expressed concerns about the long-term use of wearable devices, emphasizing the need to combine behavioral change techniques such as target setting, feedback, and rewards with other evidence-based techniques in order to use them effectively to prompt behavior changes [22].

However, consumer acceptance of wearable devices has not been as positive as expected. Many important issues that affect the ultimate adoption of wearable devices by consumers have been investigated, including the reliability [23,24] and validity of the measures that they monitor [25-27]. In addition, studies have confirmed that the key to a product’s success is the acceptance of wearable products by ordinary consumers and the comfort level of these products; product usability is an important aspect of this [28,29]. According to a definition by the International Organization for Standardization [30], usability refers to the effectiveness, efficiency, and user satisfaction rating of a product in a specific environment by a specific user for a specific purpose. It includes three aspects: effectiveness (ie, the accuracy and completeness of a goal that is achieved by a product); efficiency (ie, the effort required for a user to complete a task); and satisfaction (ie, the comfort and acceptability of a product). Usability tests and evaluations aim to make medical equipment easier, safer, and more effective and pleasant for users. A usability evaluation helps wearable devices to satisfy the requirements of the market and consumers [31].

In traditional usability studies, a thematic analysis based on heuristic evaluations, cognitive walkthroughs, think-alouds, and interviews is used; the results are then integrated together [32-34]. Although these methods are very popular, they have some limitations because results obtained in a laboratory environment can be difficult to interpret and translate into practice [35]. Questionnaires are another commonly employed method. They have been widely accepted by clinical medical informatics and consumer health informatics researchers and practitioners, and they are also widely applied in electronic health records, computerized physician order entry, and health applications [36-39]. At the same time, as a reliable, easy-to-operate, and low-cost method of usability evaluation, they have been recommended by the Agency for Healthcare Research and Quality [40]. Common questionnaire scales include the System Usability Scale (SUS) [41]; Ages and Stages Questionnaire; Computer System Usability Questionnaire; Post-Study System Usability Questionnaire [42]; Use, Satisfaction, and Ease [43]; and Web Analysis and Measurement Inventory [44].

Research has continuously established that usability evaluation and the resulting improvements in wearable device usability are critical to the wide and rapid adoption of wearable devices [45]. Research on the usability of wearable devices for health monitoring is still in the early stages. Schenkenfelder et al [46] investigated the user experience during running for three types of wearable devices (sports wristband, intelligent watch, and intelligent glasses). By analyzing feedback from 18 participants they found that the wristband and watch received higher SUS scores than the glasses. However, no significant differences between the wristband and watch were observed. Kaewkannate and Kim [47] compared the functions and features of four wearable devices (Withings Pulse, Misfit Shine, Jawbone Up24, and Fitbit Flex) and recruited seven users for objective and subjective evaluations. The Withings Pulse received the highest user acceptance, followed by the Fitbit Flex, Jawbone Up24, and Misfit Shine.

### Significance of This Study

This study has some advantages over other similar studies: it used a larger number of devices (seven different intelligent watches and wristbands), including mainstream international and domestic devices, and involved a significantly larger number of participants (388 persons, the largest sample size among similar studies). We compared the functions and features of various devices and evaluated the usability of wearable health tracking devices currently on the market using a mature and stable SUS scale. These results will help to assess the acceptance level of wearable devices and to identify the influencing factors. As there is a growing consensus that wearable devices should be developed for health applications, this study will help to examine the issues affecting their large-scale use and it will contribute to the research and development of better health applications.

## Methods

### Device Selection

The device selection was based on open market performance data from NPD (a leading global consumer and retail data supplier) and Canalys (a leading global technology market analyst with a distinct channel focus) [48-50]. Our inclusion criteria for the wearable activity trackers included (1) continuous monitoring of some kind of physical activity (eg, steps), and (2) provision of feedback via a separate mobile device or personal computer. We considered a device to be a wearable activity tracker if it contained an accelerometer and connected to a mobile platform. The device also had to be able to connect wirelessly with handheld or desktop computers, and be compatible with either Android 1.6+ or Apple’s operating system iOS 6.0+. Therefore, current mainstream wearable devices with distinct market performance were selected as the research objects. These included the Apple Watch, Samsung Gear S, Fitbit Surge, Jawbone Up3, Mi Band, Huawei Honor Band B2, and Misfit Shine. Among these devices, the Apple Watch and Samsung Gear S are flagship products from leading mobile digital gadget vendors. The Fitbit Surge, Jawbone Up3, and Misfit Shine are well-known international sports wristband brands representative of different price ranges. Finally, the Mi Band and Huawei Honor Band B2 are leading brands of sports wristband in the Chinese market.

### Questionnaire Selection

Usability refers to the effectiveness, efficiency, and user satisfaction rating of a product in a specific environment by a specific user for a specific purpose [51]. We chose to use the SUS, proposed by John Brooke in 1986, as the usability test tool. It is a simple scale based on a questionnaire and has been widely adopted in product usability evaluations. The SUS has the following advantages: (1) it is versatile and can be used to evaluate websites [52], software [53], mobile devices [54], and medical systems [55]; (2) it is a short questionnaire that is quick to answer; (3) a final score is provided with interpretation based on a well-established reference standard [56]; (4) it is free; (5) it is suitable even when applied to small samples (N<14); and (6) it has excellent reliability (0.85). Overall, the SUS is a quick and simple method for usability evaluation.

The SUS contains 10 questions based on the Likert five-point scale; questions 1, 3, 5, 7, and 9 are positive and questions 2, 4, 6, 8, and 10 are negative. The 10 questions are closely related and are employed for the comprehensive evaluation of a product. A higher SUS score indicates better product usability.

Details about the questionnaire’s design (the 10 questions) and scoring method are given in Multimedia Appendix 1.

### Subject Recruitment and Test Design

Recruitment was based on a convenience sampling method. It leveraged WeChat, the social media platform with the largest user population in China. Over a period from July 1, 2016 to October 31, 2016, the research team used their WeChat accounts to send the questionnaire link to 2180 friends (between 8 and 314 each) and various WeChat groups (approximately 20 groups) and invited their friends to forward the link. A total of 388 volunteers who had experience with the seven wearable devices were recruited.

Once the volunteers agreed to take part in the study, consent was obtained and they were invited to complete an evaluation questionnaire about their experiences with these products.

This research passed an audit of the Peking University Biomedical Ethics Committee #IRB00001052 - 16008 - Exampt, and consent was obtained from all participants.

### Data Collection

The questionnaires were distributed and the results collected via “sojump.com,” the largest free questionnaire survey platform in the world, which satisfied all the requirements for the questionnaires in this research [57]. The questionnaires were sent and completed via personal computers and mobile terminals. The final questionnaire “Comprehensive Evaluation Questionnaire for Intelligent Wearable Devices” is accessible online [58].

### Statistical Analysis

For a descriptive statistical analysis, basic information about the respondents was collected, including gender, age, education, profession, and monthly income. Then, the product attributes (eg, price, weight, and battery life) and specific functions (whether 22 functions, such as step counting, activity time, activity type identification, sleep monitoring, and expandable new features, were supported) were summarized for the seven wearable devices. Finally, the SUS scores for the seven wearable devices used by the respondents were calculated.

For the inferential statistical analysis, the relationships between the SUS scores and product attributes (product price, weight, and battery life) were explored using Pearson product moment correlation (since price data are continuous and have a normal distribution) and Spearman rank correlation coefficient (since the weight and battery life do not have a normal distribution). The correlations between the SUS score and the functions of the 22 products (eg, step counting, activity time, activity type identification, sleep monitoring, and expandable new features) were analyzed using point-biserial correlation. The strength of the correlation was assessed based on Cohen criteria: correlations less than .30 are considered small, correlations between .30 and .50 are considered medium, and correlations greater than .50 are considered strong [59,60]. Finally, to analyze the participants’ attitudes to the usability of different products, the analysis of variance (ANOVA) method was used with the brand and user demographic information. These analyses were all completed using IBM SPSS version 20 software (IBM Corp, Armonk, NY, USA).

## Results

### Comparison of Functions and Attributes of Devices

Based on the product specifications and official websites, the features and general attributes of the seven devices were summarized and compared (see Tables 1 and 2). Table 1 summarizes the functions of each device. All seven devices had very powerful functions in three major categories (activity, health, and miscellaneous) and 20 features. The Fitbit Surge had the most features, followed by the Apple Watch, Huawei Honor B2, Samsung Gear S, Jawbone UP3, Mi Band, and Misfit Shine. The Samsung Gear S had the most sports features, the Jawbone UP3 had the most health features, and the Apple Watch had the most other additional features. All the devices supported three basic features: step counting, activity timer, and distance record. The Fitbit Surge was the only device to record the number of floors climbed, the Samsung Gear S had a unique sports track recording feature, and the Huawei Honor B2 had a unique wireless earphone. The Apple Watch, Samsung Watch, Jawbone Up3, and Fitbit Surge could measure heart rate. All the devices could monitor sleep, with the exception of the Apple Watch. Table 2 lists some of the major attributes of each device. Among the seven devices, the Misfit Shine had the longest battery life of 3 months, followed by the Mi Band, Fitbit Surge, Jawbone Up3, Huawei Honor wristband, Samsung Gear S, and Apple Watch, which had a battery life of only 18 hours. The Fitbit Surge, which weighed 354 grams, was the heaviest device, followed by the Jawbone UP3, Misfit Shine, Apple Watch, Huawei Honor Band B2, Samsung Gear S, and Mi Band, which weighed only 5 grams.

### System Usability Scale Questionnaire Respondents

A total of 388 volunteers completed the questionnaire for this research. The volunteers included 83 Apple Watch users, 36 Samsung Gear S users, 37 Fitbit Surge users, 32 Jawbone Up3 users, 122 Mi Band users, 47 Huawei Honor Band B2 users, and 31 Misfit Shine users. Of the volunteers, 257 (66.2%) were male and 131 (33.8%) were female. The demographic information of the participants is shown in Table 3.

### System Usability Scale Scores for Each Device and Each Question

Based on the SUS calculation formula, the SUS scores for each brand are shown in Table 4. The mean SUS score for each question was calculated using SPSS, as shown in Table 5 (for full results, see Multimedia Appendix 2). For further visualization, please see corresponding boxplot diagrams in Multimedia Appendixes 3 and 4.

### Correlation Between System Usability Scale Score and Product Characteristics

We first explored the relationship between the SUS score and product characteristics (see Table 2). Pearson product moment correlation was used to analyze the relationship between the SUS value and the price, and Spearman rank correlation coefficient was used to analyze the relationship between the SUS values and the weight and battery life. We found a very weak correlation between the SUS score and the price of the product (*r*=–.10, *P=*.03), and no significant correlation between the SUS scores and the weight (*r*=–.04, *P*=.39) or battery life (*r*=.09, *P*=.10).

We then explored the relationship between the SUS scores and the functions supported by the products (see Table 1) using point-biserial correlation. We found that the SUS score had a very weak positive correlation (*r*=.11, *P*=.02) with the devices that supported activity type identification, and a very weak negative correlation (*r*=–.11, *P*=.02) with devices that supported expandable new features, as shown in Table 6.

### Correlation Between System Usability Scale Score, Device Brand, and Participants’ Demographic Information

To investigate a participant’s perception of the usability of each of the seven brands of wearable devices, seven ANOVA tests were performed. These were used to determine the correlation between the SUS scores, device brand, and participants’ demographic information (length of time the device was used, gender, age, education, profession, and monthly income) (see Table 7).

**Table 1:** Function summary of the seven wearable devices.

Items		Apple Watch	Samsung Gear S	Jawbone Up3	Fitbit Surge	Misfit Shine	Huawei Honor B2	Mi Band
**Activity**								
	Step counting	Yes	Yes	Yes	Yes	Yes	Yes	Yes
	Activity time	Yes	Yes	Yes	Yes	Yes	Yes	Yes
	Activity type identification	—^a^	—	Yes	Yes	Yes	Yes	Yes
	Floor climbing	—	—	—	Yes	—	—	—
	Energy consumption	Yes	Yes	Yes	Yes	Yes	Yes	Yes
	Running track	—	Yes	—	—	—	—	—
	Activity goal	Yes	Yes	Yes	Yes	Yes	Yes	Yes
	Professional fitness record	Yes	Yes	—	Yes	—	—	Yes
**Health**								
	Heart rate	Yes	Yes	Yes	Yes	—	—	—
	Sleep monitoring	—	Yes	Yes	Yes	Yes	Yes	Yes
	Diet record	—	—	Yes	Yes	Yes	—	—
	Inactivity notification	Yes	—	Yes	—	Yes	Yes	Yes
**Miscellaneous**								
	Incoming call alert	Yes	Yes	—	Yes	Yes	Yes	Yes
	Vibrating alarm clock	Yes	Yes	Yes	Yes	Yes	Yes	Yes
	Text message display	Yes	Yes	—	Yes	Yes	—	—
	Time display	Yes	Yes	—	Yes	Yes	—	—
	Remote control photograph	Yes	—	—	—	Yes	—	—
	Sports achievement sharing	Yes	—	Yes	Yes	Yes	Yes	Yes
	Bluetooth synchronization	Yes	Yes	Yes	Yes	—	Yes	Yes
	Expandable new features	Yes	Yes	—	—	—	—	—
Total		15	14	12	16	14	11	12

^a^Device does not support this feature.

**Table 2:** Major attributes of the seven wearable devices.

Device	Country	Date to market	Price (US$)	Weight (g)	Battery life (days)
Apple Watch	USA	September 10, 2014	$402	40.00	0.75
Samsung Gear S	Korea	September 25, 2013	$404	122.50	2.00
Fitbit Surge	USA	October 27, 2014	$279	354.00	7.00
Jawbone Up3	USA	November 5, 2014	$183	29.00	7.00
Mi Band	China	July 22, 2014	$12	5.00	30.00
Huawei Honor B2	China	March 1, 2015	$155	40.00	5.00
Misfit Shine	USA	September 14, 2013	$77	9.40	180.00

**Table 3:** Demographic information of the respondents (N=388).

Items		n (%)
**Device to be evaluated**		
	Apple Watch	83 (21.4)
	Samsung Gear S	36 (9.3)
	Fitbit Surge	37 (9.5)
	Jawbone Up3	32 (8.2)
	Mi Band	122 (31.4)
	Huawei Honor B2	47 (12.1)
	Misfit Shine	31 (8.0)
**Length of time the device was used**		
	<1 week	49 (12.6)
	1 week-1 month	96 (24.7)
	1-3 months	66 (17.0)
	3-6 months	53 (13.7)
	6 months-1 year	69 (17.8)
	1-2 year	49 (12.6)
	>2 years	6 (1.5)
**Gender**		
	Male	257 (66.2)
	Female	131 (33.8)
**Age**		
	Under 18	10 (2.6)
	18~25	107 (27.6)
	26~30	88 (22.7)
	31~40	113 (29.1)
	41~50	56 (14.4)
	51~60	13 (3.4)
	>60	1 (0.3)
**Education**		
	Under primary school	3 (0.8)
	Primary school-high school	19 (4.9)
	Junior college	46 (11.9)
	Bachelor	185 (47.7)
	Master of Science or Master of Arts	98 (25.3)
	PhD	37 (9.5)
**Profession**		
	Internet	114 (29.4)
	Finance	28 (7.2)
	Health care	92 (23.7)
	Manufacturing	19 (4.9)
	Fast consumer goods	4 (1.0)
	Education	29 (7.5)
	Law	6 (1.5)
	Trade	6 (1.5)
	Service	20 (5.2)
	Advertising and media	6 (1.5)
	Agriculture, forestry, fishery	2 (0.5)
	Miscellaneous	62 (16.0)
**Monthly income (US$)**		
	<145	27 (7.0)
	145~290	34 (8.8)
	291~580	45 (11.6)
	581~1160	93 (24.0)
	>1160	189 (48.7)

**Table 4:** Total System Usability Scale (SUS) score of each device (N=388).

Device	n	Mean (SD)	High score	Low score	% of total sum
Apple Watch	83	61.36 (14.69)	95.00	27.50	20.40
Samsung Gear S	36	62.08 (19.29)	100.00	5.00	9.00
Fitbit Surge	37	63.85 (21.97)	100.00	0.00	9.50
Jawbone Up3	32	65.94 (17.53)	100.00	27.50	8.50
Mi Band	122	65.12 (14.73)	100.00	35.50	31.80
Huawei Honor B2	47	67.61 (16.12)	100.00	22.50	12.70
Misfit Shine	31	65.97 (20.21)	100.00	10.00	8.20

**Table 5:** Mean System Usability Scale (SUS) score for each question and device.

Device	SUS score, mean (SD)									
	1	2	3	4	5	6	7	8	9	10
Apple Watch (n=83)	2.86 (1.00)	1.71 (1.16)	2.90 (0.89)	2.30 (1.26)	2.60 (0.95)	2.11 (1.14)	2.84 (0.89)	2.20 (1.23)	2.78 (0.96)	2.23 (1.26)
Samsung Gear S (n=36)	2.72 (1.26)	1.61 (1.29)	3.08 (1.02)	2.31 (1.37)	2.78 (0.96)	1.92 (1.27)	2.86 (1.02)	2.14 (1.44)	3.06 (0.95)	2.36 (1.31)
Fitbit Surge (n=37)	2.64 (1.38)	2.00 (1.41)	3.00 (1.07)	2.56 (1.52)	2.53 (1.06)	2.06 (1.26)	2.81 (1.17)	2.44 (1.38)	2.92 (1.08)	2.67 (1.22)
Jawbone Up3 (n=32)	2.90 (1.14)	1.90 (1.40)	2.97 (0.91)	2.58 (1.41)	2.71 (1.01)	2.16 (1.21)	2.94 (1.06)	2.55 (1.29)	3.10 (0.91)	2.42 (1.52)
Mi Band (n=122)	2.65 (1.02)	1.87 (1.30)	2.94 (1.04)	2.80 (1.33)	2.43 (1.01)	2.27 (1.13)	2.93 (1.04)	2.82 (1.11)	2.50 (1.13)	2.84 (1.19)
Huawei Honor B2 (n=47)	2.70 (1.07)	1.72 (1.19)	2.96 (1.07)	2.85 (1.28)	2.78 (0.94)	2.46 (1.21)	2.83 (0.97)	2.76 (1.21)	3.13 (0.88)	2.80 (1.13)
Misfit Shine (n=31)	2.87 (1.01)	2.33 (1.45)	3.10 (1.09)	2.87 (1.36)	2.40 (1.13)	2.27 (1.28)	2.83 (1.21)	2.60 (1.25)	2.77 (1.14)	2.50 (1.36)
Total (N=388)	2.74 (1.09)	1.84 (1.09)	2.97 (1.00)	2.61 (1.35)	2.57 (1.00)	2.20 (1.19)	2.87 (1.02)	2.54 (1.25)	2.80 (1.05)	2.58 (1.27)

**Table 6:** Point-biserial correlation analysis between the System Usability Scale (SUS) scores and product functions (N=388).

Product function	Point-biserial correlation	
	*r*	*P* value
Activity type identification	.11	.02
Expandable new features	−.11	.02
Floor climbing	.03	.58
Running track	−.04	.40
Professional fitness record	−.09	.08
Heart rate	−.09	.07
Sleep monitoring	.09	.07
Diet record	.04	.45
Inactivity notification	.01	.81
Incoming call alert	.01	.85
Text message display	−.06	.22
Time display	−.06	.22
Remote control photograph	−.05	.35
Sports achievement sharing	.04	.40
Bluetooth synchronization	.08	.13

**Table 7:** ANOVA tests of the System Usability Scale (SUS) scores of the seven devices and of the volunteer’s demographic information (profession, length of time the device was used).

Measures		Sum of squares	Degrees of freedom	Mean square	*F* (df1,df2)	*P* value
**SUS scores of devices**						
	Between groups	1671.52	6	278.59	0.98 (6,381)	.44
	Within groups	108,057.91	381	283.62	—^a^	—
	Total	109,729.43	387	—	—	—
**Profession**						
	Between groups	5618.23	11	510.75	1.84 (11,376)	.04
	Within groups	104,111.19	376	276.89	—	—
	Total	109,729.43	387	—	—	—
**Length of time device used**						
	Between groups	12,581.19	6	2096.86	8.22 (6,381)	.001
	Within groups	97,148.24	381	254.98	—	—
	Total	109,729.43	387	—	—	—

^a^Not applicable.

First, we found that there was no statistically significant difference between the SUS scores of the different brands (see Table 7), but there were significant differences in the SUS scores according to occupation and user experience (length of time the device was used). No significant difference was found for other features such as gender, age, education, and monthly income, as detailed in Multimedia Appendix 5. Second, we further used independent sample *t* tests to compare the mean SUS scores of respondents in the health care and internet industries; this indicated that the former scores were significantly higher than the latter. The SUS scores for the health care industry (n=92) were mean 68.7 (SD 14.5, range 35-100); for the internet industry (n=114) they were mean 61.1 (SD 17.9, range 5-100; *t*_204_=–3.24, *P*=.001). Third, we used Pearson product moment correlation method to explore the relationship between the SUS scores and the length of time the device was used. The SUS score had a moderate positive correlation (*r*=.32, *P*<.001) with user experience. In other words, the SUS score was related to a volunteer’s user experience (how long they had used the product). Other factors had no significant influence on the SUS score.

## Discussion

### Low System Usability Scale Scores for Seven Wearable Devices

The mean SUS score for the seven devices was 64.3 (SD 16.8). Although the Huawei Honor Band B2 received the highest score (mean 67.6, SD 16.1), based on the ANOVA analysis, there was no statistically significant difference in the mean SUS score of the seven products. Based on the SUS score, we believe that these products have little difference in terms of the ease of use. Aaron et al from AT&T Labs [61] added one question to the end of the SUS questionnaire, “Overall, I would rate the user friendliness of this product as…” This allowed users to rate the user interface using adjectives such as poor, OK, and good. The purpose of this was to associate the SUS with these adjectives. According to the data in this research, the user interfaces rated as “good” by users received a mean SUS score of 71.4. Therefore, the usability of all seven wearable devices was “good” or “OK.” The Huawei Honor Band B2, which received the highest SUS score of 67.6 (SD 16.1), was also in the “good” range, whereas the Apple Watch received the lowest score of 61.3 (SD 14.6). Although the seven brands received different SUS scores, the ANOVA test of the SUS scores versus device brands revealed no significant correlation between them. We believe that the usability of a wearable health tracking device is moderate with no significant differences between brands. These products are immature and additional breakthroughs in the core technology are required. In this sector, there is no leading brand with an absolute competitive edge, and there is no significant difference between domestic and foreign brands.

### Correlation Between System Usability Scale Score and Demographic Characteristics

The SPSS analysis indicated that the SUS scores for wearable devices are related to demographic characteristics. Factors such as user experience and profession may affect the scores. The results also revealed that user experience (the time length the device was used) could be an important factor affecting the SUS score. We found that there was a moderate positive correlation between the SUS scores and user experience (*r*=.32, *P*<.001); this matches the conclusions of MacDorman et al [62] and Kortum et al [63]. The participants in medical and health care industries gave a significantly higher score (mean 68.7, SD 14.5) than the participants in other industries, particularly those in the internet industry (mean 61.1, SD 17.9), with the former giving scores that approached the “good” level (71.4) in the acceptance diagram [61]. We conducted independent sample *t* tests to compare the mean SUS scores of participants in the health care and internet industries, which indicated that the former score was significantly better than the latter (*P*<.001). This suggests that in our sample set medical and health care employees evaluated the devices more highly and that their acceptance level of wearable device usability was higher than internet employees, indirectly showing that the demand for wearable devices is more urgent in health-related industries, a potential reason or phenomenon worthy of further research and analysis in the future. This may be due to the serious shortage of medical resources in China, especially in remote areas. The gap between supply and demand provides opportunities for mobile medical-based wearable equipment, whereas the rapid development of mobile internet and big data technologies provide the support required for the development of mobile medical treatments. In the future, patients with chronic diseases such as coronary heart disease, hypertension, and diabetes will not only receive drug treatment, but integrated disease management programs including remote monitoring, tele-treatment plan adjustments, and lifestyle management through wearable technology. Previous SUS research [61] indicated that no control was needed for gender or that gender was not a key influencing factor for consumer-grade products. Our result has validated this conclusion.

### Correlation Between System Usability Scale Score and Product Characteristics

Some characteristics of wearable devices are related to the SUS scores. Our study found that the intelligent recognition of activity type had a very weak positive correlation with the SUS score, and that expandable new functions and price have a very weak negative correlation with the SUS score. We believe that these findings, to some extent, show a “user portrait” of Chinese wearable device consumers who are likely to prefer devices that are functional and easy to operate (a weak positive correlation between the SUS score and the intelligent recognition function of the device). The price was a favorable factor, although it was not strong (the SUS score had a weak negative correlation with the price of device). We found that this is in line with the current market strategy of the Mi Band bracelet which applies the lowest price (only US $12) and the most core functions (such as steps, exercise time, sleep time, and resting time) to maximize the market share. Using this strategy, Mi Technologies Inc sold more than 3.6 million Mi Band bracelets in China in the first quarter of 2017, surpassing the giant manufacturers Fitbit, Apple, and Samsung to become the world’s largest wearable device maker [64]. In addition, we found that Q3 and Q7 (see Multimedia Appendix 1) had the highest scores: 2.97 and 2.87, respectively (see Table 5 and Multimedia Appendix 2). These scores were related to the ease of use (accessibility) of the product and the fact that people are generally more inclined to use products that do not require experience or focus and do not produce, or produce less, cognitive stress [65]. We think that this may be one reason for the high short-term usage rate of wearable devices. Although the use of wearable devices can enhance the ability to monitor the user’s behavior, if they perform similar activities and movements every day then, over time, it becomes easier to stop using the device as the user’s perception of the link between the results of the behavior intervention and the device monitoring is lost [66]. In order to prolong the use-life cycle and long-term wear rate of wearable devices, we need to integrate more cognitive behavior change strategies and functions to promote and consolidate the changes in users’ habits. This includes the identification of behavioral disorders and adjustment of cognitive attitudes [67]. At the same time, we believe that the low SUS scores of the current fitness wearable devices are due to the users’ dissatisfaction with the function and effect of the equipment, or their lack of cognitive motivation to change their behavior for a healthy lifestyle [22]. Therefore, we suggest that developers focus on the core requirements of the user, combined with social support, environmental support factors, customizable self-monitoring schemes, and personalized feedback, which should be integrated into the wearable device’s regular monitoring functions. In this way, we can help to improve the user’s overall usability evaluation of such products, prolong their use time, increase product adhesion, and cultivate user loyalty.

### Implications of Feature Analysis: Function Homogeneity, Awaiting Killer Function

Wearable devices have great potential in personal health management and clinical care. In future, the cost of wearable devices will decrease, such as the Mi Band which is sold for only US $12. Wearable devices can record sports data, help set exercise goals, and avoid excessive movement, making it easy to obtain sports performance data and maintaining and promoting the passion for sport. In addition, wearable devices can play a role in clinical practice. An obvious characteristic of wearable devices is the continuous monitoring of health data, which can provide clues for disease diagnosis, identify disease symptoms in a timely manner, and help doctors to make more comprehensive and accurate judgments.

Current mainstream wearable devices, including the seven brands in this study, are powerful; they not only track and record health data, such as sports data, sleep, and heart rate, but also provide numerous additional functions (including call notification, social media sharing, and Bluetooth). However, the function and feature analyses show that their functions are homogeneous and most concentrated on three functions: activity, reminders, and health monitoring. From this point of view, the homogenization of the product functions is very serious, and the functions of each product are lacking. There are no killer functions that solve major health management or clinical questions. Accordingly, the perceived usability of mainstream wearable devices is unsatisfactory and customer loyalty is not high. In fact, some recent studies have shown that many users use activity trackers for only a short time [68].

### Characteristics and Limitation of This Research

In this research, the features of different devices were compared and their usability evaluated using the SUS. Compared to similar studies, we investigated the most extensive range of devices and used the largest group of participants. However, this research has its limitations. First, we adopted the snowball convenient randomization method; the participants were recruited based on the research teams’ circle of WeChat friends. Although the final respondents recruited were distributed across the country, this sample is not sufficient to represent consumers of wearable devices nationwide. Nonetheless, since our sample size was large and the responses were autonomous, we believe that our group still has a certain degree of representativeness. This is reinforced by the fact that the proportion of each brand in our study (Mi 31.4%, Huawei 12.1%, and Samsung 9.3%) is similar to the market share of intelligent wristbands in the 2014 report by Tencent ISUX [69] (Mi 34.3%, Huawei 12.5%, and Samsung 10.5%). Second, the survey was conducted using online questionnaires. Although this method is more convenient, faster, and more efficient than a conventional paper questionnaire in terms of distribution, collection, and analysis, many factors are uncontrollable and some factors, such as behavioral cognitive factors [70], social network factors [71], and environmental support factors [72], may affect the results.

### Conclusions

The homogenization of wearable device functions is obvious, the usability of popular wearable devices on the market is unsatisfactory, and these devices have not been completely accepted by consumers. Usability is similar among the different brands with no absolute leader. No significant differences between domestic and international brands were observed. A consumer’s SUS score for wearable devices is related to their personal situation rather than the device brand. Device manufacturers should put more effort into developing innovative functions and improving the usability of their products.

## Appendix

Multimedia Appendix 1 System Usability Scale (SUS) questionnaire design and scoring method.

Multimedia Appendix 2 System Usability Scale (SUS) score of each question for each device.

Multimedia Appendix 3 System Usability Scale (SUS) scores for all devices.

Multimedia Appendix 4 System Usability Scale (SUS) scores for all questions.

Multimedia Appendix 5 ANOVA tests of the System Usability Scale (SUS) scores and the volunteer’s demographic information.

## Abbreviations

ANOVA: analysis of variance
SUS: System Usability Scale
