# Supplementary material for: Usability Study of Mainstream Wearable Fitness Devices: Feature Analysis and System Usability Scale Evaluation
Source: JMIR Mhealth Uhealth. 2018 Nov 8;6(11):e11066. doi: 10.2196/11066 (PMC6250954; doi:10.2196/11066)
Supplement: Multimedia Appendix 1 [file mhealth_v6i11e11066_app1.pdf]

Appendix A

A-1 SUS questionnaire design and scoring method

For the questionnaire content, ten questions are prepared based on the classical SUS questionnaire by John Brooke and suggestions from other researchers:

Q1. I think that I would like to use this product frequently;

Q2. I found the product unnecessarily complex;

Q3. I thought the product was easy to use;

Q4. I think that I would need the support of a technical person to be able to use this product;

Q5. I found the various functions in this product were well integrated;

Q6. I thought there was too much inconsistency in this product;

Q7. I would imagine that most people would learn to use this product very quickly;

Q8. I found the product very cumbersome to use;

Q9. I felt very confident using the product;

Q10. I needed to learn a lot of things before I could get going with this product.

The SUS scoring rule is as follows: (1) based on the level, each problem has a base score that ranges from 1–5, which corresponds to the range from “strongly disagree” to “strongly agree”; (2) the scores for questions 1, 3, 5, 7, and 9 are equal to the base score minus 1; the scores for questions 2, 4, 6, 8, and 10 are equal to five minus the base score; (3) the scores of the ten questions are added to obtain the total score of the

questionnaire; and the questionnaire SUS score is the total score times 2.5. The SUS is represented as a percentage.

$$SUS = 2.5 \times \left[\sum_{n=1}^{5} \left(U_{2n-1} - 1 \right) + \left(5 - U_{2n} \right) \right]$$
