# Supplementary material for: Usability Study of Mainstream Wearable Fitness Devices: Feature Analysis and System Usability Scale Evaluation
Source: JMIR Mhealth Uhealth. 2018 Nov 8;6(11):e11066. doi: 10.2196/11066 (PMC6250954; doi:10.2196/11066)
Supplement: Multimedia Appendix 2 [file mhealth_v6i11e11066_app2.pdf]

Appendix A

A-2 SUS score of each question for each device

Table 5. SUS mean score of each question for each device.

Device		SUS _1	SUS _2	SUS _3	SUS _4	SUS _5	SUS _6	SUS _7	SUS _8	SUS _9	SUS _10
AW (N=83)	Mean	2.86	1.71	2.90	2.30	2.60	2.11	2.84	2.20	2.78	2.23
	Std. Deviation	1.00	1.16	0.89	1.26	0.95	1.14	0.89	1.23	0.96	1.26
SGS (N=36)	Mean	2.72	1.61	3.08	2.31	2.78	1.92	2.86	2.14	3.06	2.36
	Std. Deviation	1.26	1.29	1.02	1.37	0.96	1.27	1.02	1.44	0.95	1.31
FS (N=37)	Mean	2.64	2.00	3.00	2.56	2.53	2.06	2.81	2.44	2.92	2.67
	Std. Deviation	1.38	1.41	1.07	1.52	1.06	1.26	1.17	1.38	1.08	1.22
JU3 (N=32)	Mean	2.90	1.90	2.97	2.58	2.71	2.16	2.94	2.55	3.10	2.42
	Std. Deviation	1.14	1.40	0.91	1.41	1.01	1.21	1.06	1.29	0.91	1.52
MB (N=122)	Mean	2.65	1.87	2.94	2.80	2.43	2.27	2.93	2.82	2.50	2.84
	Std. Deviation	1.02	1.30	1.04	1.33	1.01	1.13	1.04	1.11	1.13	1.19
HHB2 (N=47)	Mean	2.70	1.72	2.96	2.85	2.78	2.46	2.83	2.76	3.13	2.80
	Std. Deviation	1.07	1.19	1.07	1.28	0.94	1.21	0.97	1.21	0.88	1.13
MS	Mean	2.87	2.33	3.10	2.87	2.40	2.27	2.83	2.60	2.77	2.50

(N=31)	Std. Deviation	1.01	1.45	1.09	1.36	1.13	1.28	1.21	1.25	1.14	1.36
Total (N=388)	Mean	2.74	1.84	2.97	2.61	2.57	2.20	2.87	2.54	2.80	2.58
	Std. Deviation	1.09	1.29	1.00	1.35	1.00	1.19	1.02	1.25	1.05	1.27
