# Supplementary material for: Usability Study of Mainstream Wearable Fitness Devices: Feature Analysis and System Usability Scale Evaluation
Source: JMIR Mhealth Uhealth. 2018 Nov 8;6(11):e11066. doi: 10.2196/11066 (PMC6250954; doi:10.2196/11066)
Supplement: Multimedia Appendix 3 [file mhealth_v6i11e11066_app3.pdf]

Appendix A

A-3 SUS scores for all devices

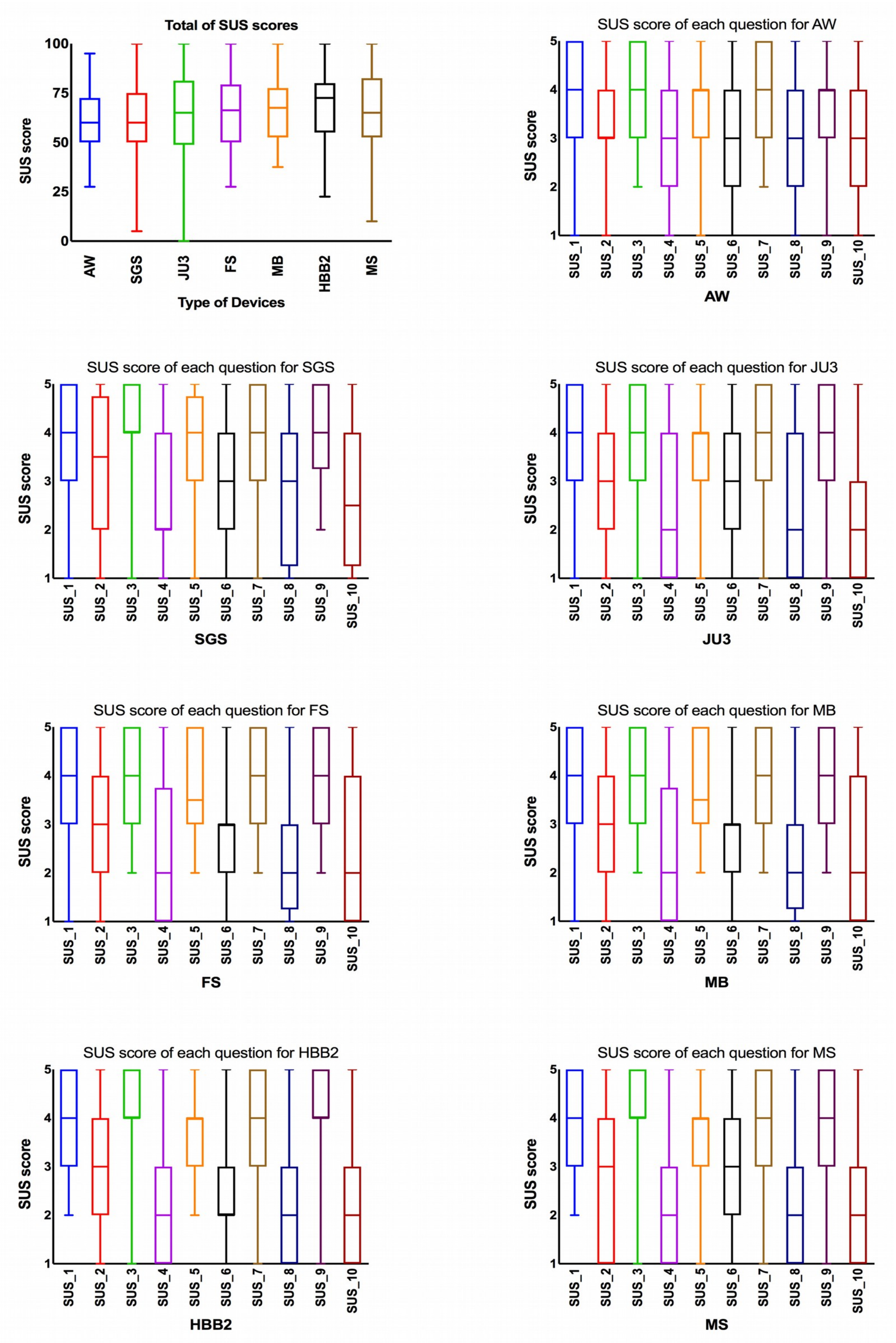

Note:

AW: Apple Watch; SGS: Samsung Gear S; FS: Fitbit Surge; JU3: Jawbone Up3; MB:

Mi Band; HHB2: Huawei Honor B2; MS: Misfit Shine
