# Supplementary material for: Usability Study of Mainstream Wearable Fitness Devices: Feature Analysis and System Usability Scale Evaluation
Source: JMIR Mhealth Uhealth. 2018 Nov 8;6(11):e11066. doi: 10.2196/11066 (PMC6250954; doi:10.2196/11066)
Supplement: Multimedia Appendix 5 [file mhealth_v6i11e11066_app5.pdf]

Appendix A

A-5 ANOVA tests of the SUS scores and the volunteer's demographic information

Table 7. ANOVA analysis of SUS scores among volunteer's demographic information (length of time the device was used, gender, age, education, profession and monthly income)

	Profession				
	Sum of Squares	df	Mean Square	F	Sig.
Between Groups	5618.23	11	510.75	1.84	.04
Within Groups	104111.19	376	276.89		
Total	109729.43	387			

	Length of Time the Device was Used				
	Sum of Squares	df	Mean Square	F	Sig.
Between Groups	12581.19	6	2096.86	8.22	<.001
Within Groups	97148.24	381	254.98		
Total	109729.43	387			

	Gender				
	Sum of Squares	df	Mean Square	F	Sig.
Between Groups	718.84	1	718.849	2.55	.11
Within Groups	109010.59	386	282.419		

Total	109729.439	387			

	Age				
	Sum of Squares	df	Mean Square	F	Sig.
Between Groups	1368.15	6	228.03	.80	.57
Within Groups	108361.28	381	284.41		
Total	109729.43	387			

	Education				
	Sum of Squares	df	Mean Square	F	Sig.
Between Groups	2671.30	5	534.26	1.91	.092
Within Groups	107058.13	382	280.26		
Total	109729.43	387			

	Monthly Income				
	Sum of Squares	df	Mean Square	F	Sig.
Between Groups	2629.02	4	657.26	2.35	.06
Within Groups	107100.41	383	279.64		
Total	109729.43	387			
